# Multiphase antibiofilm potential of shrimp-shell–derived chitosan nanoparticles against Aeromonas hydrophila isolated from tropical aquaculture environments

**DOI:** 10.14202/vetworld.2025.3870-3887

**Published:** 2025-12-13

**Authors:** Rozi Rozi, Wiwiek Tyasningsih, Jola Rahmahani, Eduardus Bimo Aksono Herupradoto, Muchammad Yunus, Mohammad Anam Al Arif, Suryo Kuncorojakti, Putri Desi Wulan Sari, Annas Salleh, Suwarno Suwarno

**Affiliations:** 1 Veterinary Science Doctoral Study Program, Faculty of Veterinary Medicine, Universitas Airlangga, Surabaya, Indonesia; 2 Department of Aquaculture, Faculty of Fisheries and Marine, Universitas Airlangga, Surabaya, Indonesia; 3 Department of Microbiology, Faculty of Veterinary Medicine, Universitas Airlangga, Surabaya, Indonesia; 4 Department of Basic Veterinary Medicine, Faculty of Veterinary Medicine, Universitas Airlangga, Surabaya, Indonesia; 5 Department of Veterinary Parasitology, Faculty of Veterinary Medicine, Universitas Airlangga, Surabaya, Indonesia; 6 Department of Farm, Faculty of Veterinary Medicine, Universitas Airlangga, Surabaya, Indonesia; 7 Department of Veterinary Anatomy, Faculty of Veterinary Medicine, Universitas Airlangga, Surabaya, Indonesia; 8 Department of Veterinary Laboratory Diagnosis, Faculty of Veterinary Medicine, Universiti Putra Malaysia, Serdang, Malaysia

**Keywords:** Chitosan nanoparticles, *Aeromonas hydrophila*, biofilm inhibition, planktonic suppression, aquaculture biocontrol, One Health, circular bioeconomy

## Abstract

**Background and Aim:**

Biofilm-forming *Aeromonas hydrophila* represents a critical constraint in aquaculture, driving recurrent infections, environmental persistence, and antimicrobial resistance. Sustainable alternatives to antibiotics are urgently needed. This study evaluated the multiphase antibiofilm activity of chitosan nanoparticles (ChNPs) synthesized from *Litopenaeus vannamei* shrimp shells against clinical *A. hydrophila* isolates from Indonesian gourami (*Osphronemus gouramy*), focusing on their effects during biofilm adhesion, planktonic proliferation, and mature biofilm degradation.

**Materials and Methods:**

Between February 2024 and March 2025, diseased gourami were sampled from aquaculture sites in Surabaya, Indonesia. Three wild-type *A. hydrophila* isolates (A1G1, A2G1, A3G1) were confirmed via biochemical and 16S rRNA sequencing. ChNPs were synthesized through ionic gelation of deacetylated chitosan with sodium tripolyphosphate and characterized by Scanning Electron Microscopy (SEM), dynamic light scattering, and Fourier Transform Infrared Spectroscopy (FTIR) analyses. Antibiofilm efficacy was tested at concentrations of 15–45 µg mL^-¹^ using crystal violet staining (optical density [OD]_595_) for adhesion and degradation phases, and turbidity (OD_600_) for planktonic inhibition. Data were analyzed using one- and two-way analysis of variance with Tukey’s post hoc test.

**Results:**

ChNPs exhibited spherical morphology (≈641 nm; ζ = +51 mV) and stable ionic crosslinking. They significantly inhibited adherent biomass formation (p < 0.05), reducing OD_595_ from 0.787 to 0.317 in the most responsive strain A3G1 (> 59 % inhibition). Planktonic growth (OD_600_) declined dose-dependently (63 % inhibition at 45 µg mL^-¹^), with significant strain–concentration interactions (p < 0.01). Mature biofilm degradation reached 63% at 45 µg mL^-¹^, approaching the level of the antibiotic-treated control. SEM and FTIR data supported electrostatic disruption and extracellular polymeric substance penetration as probable mechanisms.

**Conclusion:**

Shrimp-shell–derived ChNPs effectively suppressed *A. hydrophila* biofilms at multiple developmental stages, demonstrating a potent, biodegradable alternative for the control of aquaculture pathogens. Their integration into eco-friendly, antibiotic-free disease management aligns with circular bioeconomy and One Health frameworks. Further *in vivo* validation and formulation optimization are warranted.

## INTRODUCTION

Aquaculture is a cornerstone of global food security, yet it remains highly susceptible to infectious diseases caused by opportunistic pathogens such as *Aeromonas hydrophila* [1–5]. A major virulence mechanism of *A. hydrophila* lies in its capacity to form multicellular biofilms embedded in extracellular polymeric substances, which promote bacterial persistence, immune evasion, and increased antibiotic resistance [6–10]. In Indonesia, *A. hydrophila* has been frequently recovered from diseased gourami (*Osphronemus gouramy*) [11–13], where it is responsible for motile *Aeromonas* septicemia, a condition marked by hemorrhages, ulceration, and high mortality [2, 4, 5, 14, 15]. Although the pathogen is well recognized, detailed investigations into its biofilm-forming characteristics under tropical aquaculture conditions remain scarce. The exceptional resilience of *Aeromonas* biofilms to conventional antimicrobials has stimulated growing interest in nanoparticle-based therapeutic alternatives [16–18].

Among these, chitosan nanoparticles (ChNPs) have attracted considerable attention due to their biocompatibility, biodegradability, and potent antimicrobial mechanisms. Their cationic surface enables electrostatic interactions with the negatively charged bacterial envelope, leading to membrane destabilization, cytoplasmic leakage, and disruption of quorum-sensing networks [8–10, 19–23]. However, most prior studies on ChNPs have focused on standard laboratory strains or human pathogens, thereby limiting their relevance to aquatic systems.

This study uniquely explores the antibiofilm activity of shrimp-shell–derived ChNPs against clinical, wild-type *A. hydrophila* isolated from Indonesian gourami. It emphasizes both ecological and methodological novelty by assessing efficacy across three key biofilm stages, initial adhesion, planktonic proliferation, and mature biofilm degradation. This triphasic, aquaculture-specific framework, combined with the circular bioeconomy concept of shrimp-shell valorization, distinguishes the present research from earlier single-phase or laboratory-strain investigations, thereby addressing critical knowledge gaps in biofilm dynamics in tropical fish-pathogen systems [10, 24–26].

Despite extensive characterization of *A. hydrophila* as an opportunistic fish pathogen, most existing studies have primarily focused on its antibiotic resistance patterns, virulence gene diversity, and general pathogenic mechanisms. In contrast, relatively few investigations have explored the biofilm-forming behavior of wild-type *A. hydrophila* strains isolated from tropical aquaculture systems, particularly under environmental and host-specific conditions reflective of Southeast Asian aquaculture. Current evidence is largely derived from standard laboratory strains or temperate isolates, which may not accurately represent the adaptive physiology or environmental resilience of tropical field strains. Moreover, comprehensive evaluations of biofilm inhibition across multiple developmental phases, from initial adhesion to mature biofilm degradation, remain scarce. Most prior reports examine only one stage of biofilm dynamics, neglecting the transitional processes critical for pathogen persistence and transmission.

At the same time, the application of biopolymer-based nanoparticles such as chitosan has demonstrated promising antimicrobial properties, yet their systematic antibiofilm evaluation against aquaculture-relevant pathogens remains poorly documented. Even fewer studies have investigated shrimp-shell–derived ChNPs as a sustainable intervention within a circular bioeconomy framework, linking aquaculture waste valorization with disease control. The absence of integrated physicochemical, microbiological, and phase-specific assessments has created a significant gap in understanding how nanochitosan formulations interact with fish-pathogenic *A. hydrophila* under real-world tropical aquaculture conditions.

This study was designed to evaluate the multiphase antibiofilm efficacy of shrimp-shell–derived ChNPs against clinical, wild-type *A. hydrophila* isolates obtained from diseased gourami (*O. gouramy*) cultured in Indonesian aquaculture systems. Specifically, the study aimed to:

1. Isolate and molecularly characterize *A. hydrophila* strains from naturally infected gourami to confirm their identity and phylogenetic relatedness to global reference strains.
2. Synthesize and characterize ChNPs from *Litopenaeus vannamei* shrimp shells using ionic gelation, and analyze their morphology, particle size, and functional groups (scanning electron microscopy [SEM], dynamic light scattering [DLS], and Fourier transform infrared [FTIR] spectroscopy).
3. Assess the inhibitory effects of ChNPs across three biofilm developmental phases: initial adhesion (adherent biomass), planktonic proliferation (cell growth), and mature biofilm degradation, using optical density(OD)-based assays.
4. Determine strain-dependent differences and concentration-dependent interactions between ChNP exposure and antibiofilm responses through statistical modeling.
5. Contextualize the findings within sustainable aquaculture and One Health frameworks, highlighting the dual role of shrimp-shell waste recycling and antimicrobial resistance mitigation.

Through these objectives, the study seeks to establish a mechanistic, eco-friendly approach to controlling biofilm-associated *A. hydrophila*, providing a scientific foundation for future *in vivo* validation and formulation development aimed at antibiotic-free health management in aquaculture.

## MATERIALS AND METHODS

### Ethical approval

All experimental procedures involving aquatic animals and bacterial isolates were conducted in accordance with institutional, national, and international guidelines for the ethical treatment of research animals and biosafety management. The study protocol, including sample collection, handling of diseased fish, and processing of bacterial isolates, was reviewed and approved by the Animal Care and Use Committee of Universitas Brawijaya, Indonesia, under approval number 170-KEP-UB-2024.

Sampling and euthanasia procedures adhered to the ethical standards outlined by the Indonesian Ministry of Agriculture Regulation No. 95/Permentan/OT.140/9/2013 concerning animal welfare, and complied with the Organisation for Economic Co-operation and Development (OECD) Guidelines for the Testing of Chemicals (Section 203: Fish Acute Toxicity) and the European Directive 2010/63/EU on the protection of animals used for scientific purposes. Diseased gouramispecimens were humanely euthanized using buffered tricaine methane sulfonate (MS-222, 500 mg/L, pH 7.0–7.5) to minimize stress and pain prior to sample collection.

All bacterial isolation and nanoparticle handling were performed in a biosafety level 2 laboratory under aseptic conditions, in accordance with the institutional biosafety and biosecurity framework. No experimental infections or deliberate pathogen exposures were performed on live fish. The study used naturally infected specimens obtained from local aquaculture ponds and markets, ensuring that no additional harm or suffering was induced beyond diagnostic sampling.

All researchers involved in the project completed certified training in animal welfare, bioethics, and laboratory biosafety before initiating experimental work. Data collection, sample storage, and disposal of biological waste followed the approved institutional standard operating procedures and were overseen by the biosafety officer of the Faculty of Fisheries and Marine, Universitas Airlangga, Surabaya, Indonesia.

The research complied with the principles of the 3Rs (Replacement, Reduction, and Refinement) and with the Animal Research: Reporting of *In Vivo* Experiments 2.0 guidelines for reporting animal research. Ethical oversight ensured that all animal use was justified and necessary, and that it was conducted with maximal consideration of welfare and environmental safety.

### Study period and location

This study was conducted from February 2024 to March 2025 at the Department of Aquaculture, Faculty of Fisheries and Marine, Universitas Airlangga, Surabaya, Indonesia. Sampling was performed at various sites in Surabaya City, East Java Province (7°15′S, 112°45′E), including ornamental fish shops, traditional fish markets, and aquaculture ponds where diseased gourami exhibiting clinical symptoms were identified and collected.

### Isolation and identification of bacteria

#### Sampling design

Sampling was conducted over three visits between March and June 2024 in traditional markets across Surabaya, Indonesia (approx. −7.25, 112.75) to capture temporal variation. The sample size (n = 90 gourami) was determined to detect an expected prevalence of 25%–30% with ±10% precision at 95% confidence. Fish were handled following ethical guidance; when required, euthanasia was performed using buffered MS-222 (tricaine) at 500 mg/L, adjusted to physiological pH (7.0–7.5) with sodium bicarbonate.

#### Primary isolation

Swabs from the kidney and intestinal tracts were transported on ice (4°C) and processed within ≤ 6 h of collection (backup storage ≤ 24 h at 4°C). Swabs were streaked aseptically onto MacConkey agar (Oxoid, UK) and Glutamate Starch Phenol Red (GSP) agar (Oxoid), followed by incubation at 32 ± 2 °C for 24–48 h. Colonies presenting yellow centers on GSP and negative lactose fermentation on MacConkey agar were considered presumptive *Aeromonas* spp. and subsequently purified on Tryptic Soy Agar (TSA; Oxoid). Pure cultures were stored in Todd-Hewitt broth (Oxoid) supplemented with 20% glycerol at −20°C and lyophilized using a 7.5% glucose–horse-serum cryoprotectant. Preliminary screening based on colony morphology, Gram staining, and Sulfide Indole Motility (SIM; Oxoid) confirmed that the isolates were motile, Gram-negative rods.

#### Biochemical characterization

Comprehensive biochemical profiling was conducted following a modified protocol from Bergey’s Manual of Determinative Bacteriology (7th ed.) [27], including oxidase and catalase activity, H_2_S production, indole, MR, Voges–Proskauer (VP), citrate utilization, gelatin hydrolysis, and urease activity (all reagents from Oxoid). Carbohydrate fermentation tests employed glucose, lactose, mannitol, sucrose, arabinose, and inositol (Oxoid), while amino acid decarboxylation was evaluated for ornithine and lysine (Oxoid).

Additional diagnostic tests included bile-esculin hydrolysis (Oxoid), ampicillin resistance, and O/129 susceptibility (Oxoid). The biochemical profiles of each isolate were systematically compared with those of the reference strain as described by Altwegg *et al*. [28], confirming phenotypic identification based on standard diagnostic characteristics. Based on their phenotypic congruence with the reference strain, we selected three wild-type isolates: A1G1, A2G1, and A3G1 for molecular characterization.

### Molecular detection of *A. hydrophila* using the *16S rRNA* gene

Following biochemical confirmation, four *A. hydrophila* isolates (ATCC 19570, A1G1, A2G1, and A3G1) were subjected to molecular identification by polymerase chain reaction (PCR) targeting the *16S rRNA* gene. Bacteria were cultured on TSA and grown in 50 mL TSB at 30°C for 24 h in a shaking incubator. Genomic DNA was extracted using the G-Spin™ Genomic DNA Extraction Kit (iNtRON Biotech®, Korea; Cat. No. 17121). with a modified protocol involving cell lysis in G buffer, heat incubation at 60°C, and sequential washing with buffers A and B, followed by elution in 200 µL elution buffer and storage at −20°C.

PCR amplification used universal primers 27F and 1492R in a 50 µL reaction containing GoTaq Green Master Mix (Promega, USA), with cycling conditions of initial denaturation (95°C, 5 min), followed by 30 cycles of denaturation (94°C, 30 s), annealing (55°C, 30 s), extension (72°C, 1 min), and a final extension at 72°C for 7 min. Amplicons were separated by electrophoresis on a 1% agarose gel pre-stained with FluorVue™ stain (SMOBIO Technology, Inc., Taiwan) and visualized under blue light or ultra-violet transilluminator (Bio-Rad Laboratories, USA); the ~1,500 bp band indicated successful 16S rRNA amplification.

Positive samples were sequenced using the Sanger method, aligned with Multiple sequence alignment was performed using ClustalW (https://www.genome.jp/tools-bin/clustalw) implemented in MEGA version 12 (https://www.megasoftware.net)., and analyzed using BLAST for species confirmation. Phylogenetic analysis was conducted using the maximum likelihood (ML) method with 1,000 bootstrap replicates under the Tamura–Nei model with invariant sites (TN93+I), selected based on the lowest BIC, AICc, and highest log-likelihood. All sites, NJ/MP initial trees, and the nearest-neighbor-interchange algorithm were used for tree inference.

### Synthesis of chitosan

ChNP was synthesized by ionotropic gelation as described by Ikono *et al*. [8], using low-molecular-weight, biocompatible-grade chitosan (degree of deacetylation [DD] = 97.8 ± 0.2%, n = 3). Chitosan (0.3 g) was dissolved in 100 mL of 0.1 M acetic acid, adjusted to pH 3.5, and magnetically stirred for 24 h. An aqueous TPP solution (0.25% w/v) was gradually added under continuous stirring (900 rpm, 60 min, 25°C ± 2°C) to yield a chitosan: TPP mass ratio of 6:1 (w/w); gelation occurred within ~20 min. After synthesis, the suspensions were washed/centrifuged to reduce residual acetic acid/TPP, sterile-filtered (0.22 µm), and used for biological testing. Working suspensions were prepared in TSB + 1% sucrose at concentrations of 15, 30, and 45 µg mL^-¹^. Concentrations are reported as mass per volume and were pre-selected as sub-inhibitory based on preliminary range-finding (data not shown) to enable comparative antibiofilm evaluation without reducing overall growth. Particle size and PDI (DLS), ζ-potential (mV), FTIR features, and inter-batch size variability are provided in the Characterization subsection.

### Characterization of the ChNP

Particle size and dispersity were measured by DLS; Delsa™ Nano Zeta Potential Analyzer, Beckman Colter, New Zealand) in water at 25 °C (η = 0.8878 cP), reporting Z-average and polydispersity index (PDI); batches with PDI > 0.30 were discarded. The zeta potential (ζ) was determined on the same instrument by electrophoretic light scattering (Smoluchowski model) under identical conditions; batches with c| < 25 mV were excluded. Morphology was examined by SEM (~20,000×, 5 kV; sputter-coated where applicable) following standard preparation. FTIR-ATR (IRTracer-100, Shimadzu Corp., Kyoto, Japan); 4000–400 cm^-¹^, 4 cm^-¹^ resolution, 32 scans) was used to verify functional-group signatures (O–H/N–H, amide, P=O) consistent with TPP crosslinking. The DD% of the chitosan precursor was determined by the FTIR peak-ratio method (A1655/A3450) with calibration (or by potentiometric titration, as applicable). Batch yield was calculated as (mass of recovered dried ChNP/initial chitosan) × 100%.

### Biofilm inhibition assays

This study employed a completely randomized design (CRD) with four treatments of ChNP concentration: 0 μg/mL (0%) (untreated control), 15 μg/mL (15%), 30 μg/mL (30%), and 45 μg/mL (45%). Each treatment comprised five technical replicates (five wells per assay) and was repeated in three independent biological runs on separate days (total N = 15 wells per treatment per strain) for four *A. hydrophila* strains, reference strain ATCC 19570, and three wild-type isolates (A1G1, A2G1, A3G1) from diseased gourami, Surabaya, Indonesia.

ChNP levels were chosen after solubility optimization and preliminary inhibition screening to represent a biologically relevant, non-cytotoxic range. Controls were explicitly differentiated: negative (untreated), vehicle (acetic acid matrix without chitosan) to exclude solvent effects, and positive (reference antibacterial/disinfectant applied at its *in vitro* labeled dose and exposure, as specified in the Results section), defining the expected maximal inhibitory response. Statistical analyses were performed using the procedures and software detailed in the Statistics subsection (α = 0.05). Nanoparticle characterization confirmed ζ-potential: 51 mV by electrophoretic light scattering, consistent with colloidal stability and electrostatic antibacterial interactions. The chitosan feedstock’s DD% is reported in the Supplement, and batch performance showed low variation in hydrodynamic size and consistent synthesis yield from shrimp-shell–derived material.

### Planktonic cell growth inhibition (OD600 nm)

A turbidity-based microdilution assay was performed to evaluate the inhibitory effect of chitosan nanoparticles (ChNPs) on the free-living (planktonic) growth phase of *A. hydrophila*. Overnight cultures of *A. hydrophila* were grown in Luria–Bertani (LB) broth (Oxoid) at 30°C with continuous shaking at 180 rpm. Subsequently, the cultures were diluted to an initial OD of 0.05 at 600 nm (OD600) in fresh LB medium. A total of 200 µL of this bacterial suspension was transferred into sterile 96-well flat-bottom polystyrene microplates, and various sub-inhibitory concentrations of ChNPs were added. Wells without treatment were used as negative controls. The microplates were statically incubated at 30°C for 24 h, after which the OD was measured at 600 nm using a microplate spectrophotometer. A reduction in OD600 compared with the untreated control indicated inhibition of planktonic cell growth.

Negative controls consisted of untreated biofilms; vehicle controls contained the acetic acid/TPP matrix matched to the highest ChNP dilution; and gentamicin at 10 µg/mL for 24 h served as the positive reference, defining maximal biofilm degradation under *in vitro* conditions. Unless otherwise stated, statistical comparisons were performed with the vehicle control. The percentage inhibition was calculated using the following formula:


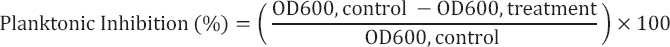


Where OD control corresponds to the CV-stained biomass in wells without ChNPs, and OD treatment refers to the stained biomass in wells treated with ChNPs.

### Quantification of the initial adherent biofilm biomass (OD595 nm)

To quantify the initial adhesion phase of biofilm formation, a crystal violet staining method was used. *A. hydrophila* overnight cultures were diluted to OD600 = 0.05 in LB broth, and 200 µL of this suspension was seeded into 96-well polystyrene microplates containing ChNPs at various concentrations. Plates were incubated at 30°C without shaking for 24 h to facilitate surface attachment. After incubation, the wells were gently washed three times with phosphate-buffered saline (PBS; pH 7.4) to remove non-adherent cells. The remaining surface-attached biomass was fixed with absolute methanol for 15 min, air-dried at 25°C ± 2°C., and stained with 0.5% (w/v) crystal violet solution (Sigma-Aldrich, St. Louis, MO, USA) for 10 min. The unbound dye was rinsed off with distilled water, and the retained stain was solubilized with 33% (v/v) glacial acetic acid. The absorbance of the eluted solution was then measured at 595 nm (OD595) using a microplate reader. OD595 from the CV assay was used as a relative biomass index and normalized to the untreated control; conversion to an absolute biomass calibration curve was not performed, consistent with standard CV-based comparative workflows. The percentage of inhibition of initial biofilm formation was calculated using the following formula:


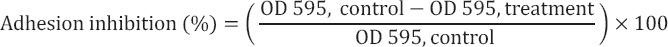


Where OD595_control corresponds to CV-stained biomass in wells without ChNPs, and OD595_treatment

refers to CV-stained biomass in ChNP-treated wells.

### Biofilm degradation assay (OD595 nm)

A biofilm degradation assay was conducted to assess the ability of ChNPs to disrupt mature biofilms, following Rivera *et al*. [8] with modifications for *A. hydrophila*. Bacterial cultures were diluted to OD600 = 0.05, 200 µL of the suspension was dispensed into sterile 96-well polystyrene microplates. Plates were incubated statically at 30°C for 48 h to allow biofilm maturation. The supernatant was gently discarded, and the wells were washed three times with PBS to remove residual planktonic cells. Fresh LB containing ChNPs at the desired concentrations was added (200 µL/well), and the plates were preincubated at 30°C for 24 h. Accordingly, ChNP treatment levels (15, 30, and 45 µg/mL) were selected from prior solubility optimization and preliminary inhibition screening to bracket a biologically relevant, non-cytotoxic window. Negative controls consisted of untreated bacterial suspensions, vehicle controls used an acetic acid/TPP matrix matched to the highest ChNP dilution, and gentamicin 10 µg/mL for 24 h served as the positive reference; unless otherwise stated, statistical comparisons were performed against the vehicle control.

Each assay included clearly differentiated controls, negative (untreated), positive (gentamicin 10 µg; Sigma-Aldrich, USA), and vehicle (acetic-acid matrix without chitosan, matched to the solvent composition of ChNPs), to attribute effects specifically to ChNPs. Replication was prespecified as five technical wells per treatment within a CRD, with the entire assay repeated as three independent biological runs on separate days; data analysis followed the procedures and software detailed in the Statistics subsection (α = 0.05). The wells were washed to remove debris and non-adherent remnants following treatment. The remaining biofilm matrix was fixed with methanol for 15 min, air-dried, and stained with 0.5% crystal violet for 10 min at 25°C ± 2°C. Excess dye was removed with distilled water, and bound CV was eluted with 33% (v/v) glacial acetic acid. The OD was measured at 595 nm (± 5 nm) using a Thermo Scientific Multiskan FC microplate photometer (USA). The percentage of biofilm degradation was determined using the following general formula:


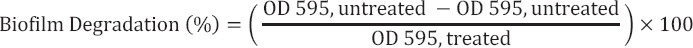


Where, OD_595_ untreated, denotes the CV-stained mature biofilm without ChNP treatment, and OD_595_ treated, denotes the residual biofilm after ChNP exposure.

### Statistical analysis

All analyses were performed using IBM SPSS Statistics version 25.0 (IBM Corp., Armonk, NY, USA) and GraphPad Prism version 10.4.2 (GraphPad Software, San Diego, CA, USA). Data are presented as mean ± standard deviation (SD) from n = 3 independent biological replicates, each comprising 5 technical replicates per treatment. The Shapiro–Wilk and Levene tests were used to verify data normality and variance homogeneity. When assumptions were violated, the data were transformed (log or Box–Cox) or analyzed using Welch’s analysis of variance (ANOVA) followed by Games–Howell post hoc tests, yielding consistent conclusions. One-way ANOVA followed by Tukey’s Honestly Significant Difference (HSD) was used to assess treatment effects within each strain, whereas two-way ANOVA was used to evaluate the interaction between strain type and ChNP concentration, particularly in planktonic growth inhibition and biofilm degradation assays. For all analyses, we report F(df_1_, df_2_), exact p-values, and effect sizes (partial η²) with 95% confidence interval (CI) wherever applicable. Statistical significance was set at α = 0.05 (a two-tailed Student’s *t*-test). This study generated no new regression or modeling data; however, predictive modeling (dose–response or nonlinear regression) and structure–function correlation analyses (PCA) are identified as future analytical extensions to refine inhibition kinetics and nanoparticle–biofilm relationships.

## RESULTS

### Biochemical characterization of *A. hydrophila* isolates

Four *A. hydrophila* strains were used in this study, including a reference strain (ATCC 19570) and three wild-type isolates (A1G1, A2G1, and A3G1) obtained from diseased gourami collected in Surabaya, Indonesia. Based on Table 1, three bacterial isolates (A1G1, A2G1, and A3G1) underwent comprehensive biochemical profiling and were compared against the reference characteristics of *A. hydrophila*, as described by Altwegg *et al*. [27].

**Table 1:** Biochemical characteristics of *A. hydrophila* isolates (A1G1, A2G1, and A3G1) compared with the reference strain [27].

Biochemical tests	A1G1	A2G1	A3G1	Altwegg *et al.* [27]
Gram-stain	–	–	–	–
Catalase	+	+	+	+
Oxidase	+	+	+	+
H_2_S	–	–	–	–
Indol	+	+	+	+
Metyl-red	–	–	–	–
Voges-Proskaeur	+	+	+	+
Simon citrate	–	+	–	–
Lysine dekarboksilase	–	+	+	–
Ornithine decarboxylation	–	–	–	–
Gelatin	+	+	+	+
Urea	+	+	+	+
Aesculin	+, gas	+, gas	+, gas	+, gas
Glucose	+	+	+	+
Sucrose	+	+	+	+
Lactose	V	V	V	V
Arabinose	+	+	+	+
Mannitol	V	V	V	V
Inositol	+	+	+	–
Ampicillin	R	R	R	R
O/129	+	+	+	+
Percentage similarity	95.23%	85.27%	90.47%	

All isolates exhibited Gram-negative reactions and were positive for catalase, oxidase, indole, Voges–Proskauer, gelatin hydrolysis, urea hydrolysis, aesculin fermentation (with gas production), glucose, sucrose, and arabinose fermentation. Negative reactions were observed for hydrogen sulfide production, methyl red, and ornithine decarboxylation across all isolates.

Distinct inter-isolate variations were observed. For instance, A2G1 was the only isolate that showed positive results for both citrate utilization and lysine decarboxylation, whereas A1G1 and A3G1 were negative for citrate utilization; lysine decarboxylase activity was observed only in A2G1 and A3G1. Inositol fermentation was positive in all isolates but absent in the reference profile. Lactose and mannitol fermentation yielded variable results across isolates and reference strains, consistent with known heterogeneity in carbohydrate metabolism among *Aeromonas* spp.

All isolates exhibited resistance to ampicillin and susceptibility to O/129 vibriostatic compound, supporting their taxonomic placement within the *A. hydrophila* complex. Based on the overall biochemical patterns, similarity indices relative to the reference profile of Altwegg *et al*. [27] were calculated as 95.23% for A1G1, 85.27% for A2G1, and 90.47% for A3G1. These results affirm species identity and indicate minor phenotypic divergence likely attributable to environmental adaptation or intra-species variability.

### Molecular identification and phylogenetic analysis based on the *16s rRNA* gene

PCR amplification of the *16S rRNA* gene from three local isolates (A1G1, A2G1, and A3G1) yielded amplicons of approximately 1,500 bp. BLAST analysis revealed high sequence similarity (99.12%–99.70%) with *A. hydrophila* strains from Turkey, Argentina, Brazil, China, India, Japan, Egypt, Greece, and South Korea.

**Figure 1:**
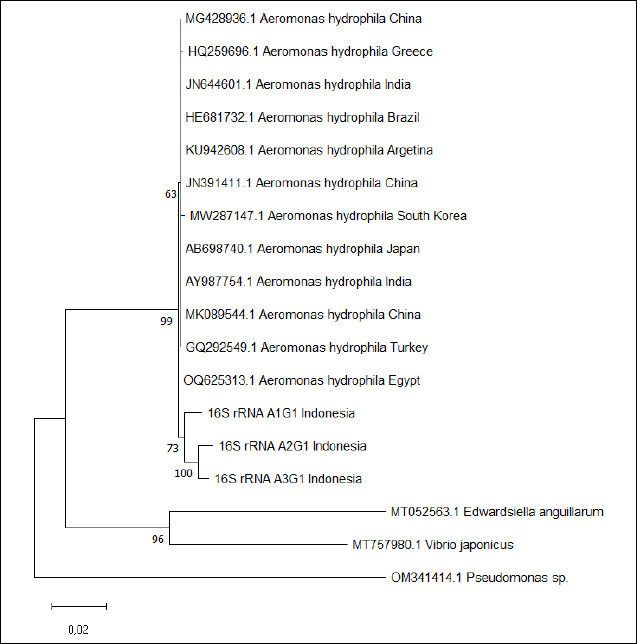
Phylogenetic tree based on 16S rRNA gene sequences of *Aeromonas hydrophila* isolates (A1G1, A2G1, and A3G1) and global reference strains retrieved from GenBank. The tree was constructed using the Maximum Likelihood method with the Tamura-Nei + I model in MEGA 12, with 1,000 bootstrap replicates. The Indonesian isolates were clustered with the reference strains from Turkey (GQ292549.1), Argentina (KU942608.1), and China (MK089544.1), while the outgroups were *Edwardsiella anguillarum* (MT052563.1)*, Vibrio japonicus* (MT757980.1), and *Pseudomonas sp*. (OM341414.1).

Phylogenetic analysis was performed using the ML method in MEGA 12 with 1,000 bootstrap replicates under the Tamura–Nei model with invariant sites (TN93+I). The resulting tree (Figure 1) showed that all Indonesian isolates clustered within the *A. hydrophila* clade, closely related to global reference strains such as GQ292549.1 (Turkey), KU942608.1 (Argentina), and HE681732.1 (Brazil), with high bootstrap support ranging from 73% to 100%. The cluster containing A1G1, A2G1, and A3G1 was clearly separated from outgroup species such as *Vibrio japonicus* (MT757980.1) and *Edwardsiella anguillarum* (MT052563.1), confirming the taxonomic position of the isolates within *A. hydrophila*.

### SEM analysis of synthesized ChNPs

**Figure 2:**
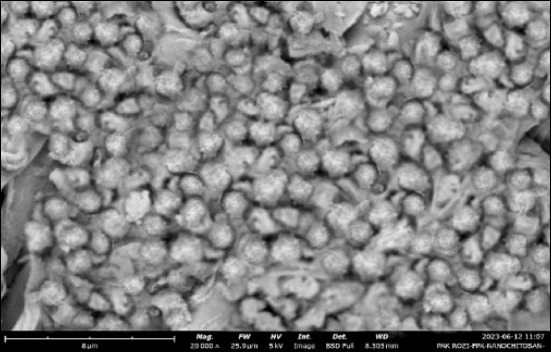
Scanning electron micrograph of nanochitosan synthesized from *Litopenaeus vannamei* shrimp shells. Spherical particles with dense and compact clustering are visible at 20,000× magnification. Scale bar: 5 μm.

A scanning electron microscope (SEM; JSM-6360, JEOL Ltd., Tokyo, Japan) was used to characterize the morphology of ChNPs synthesized from *L. vannamei* shells via ionic gelation. As shown in Figure 2, the particles exhibited predominantly spherical shapes with smooth surfaces and minimal aggregation, forming compact and homogeneous clusters. This morphology reflects effective electrostatic stabilization through ionic crosslinking between the chitosan and tripolyphosphate amine groups.

The SEM images (20,000× magnification, 5 kV) revealed a mean particle diameter of 641.5 ± 11.08 nm, consistent with nanoscale distributions reported for similar gelation-based methods. These structural features indicate a high surface-area-to-volume ratio, which supports potential applications in antimicrobial delivery, biofilm inhibition, and aquaculture therapeutics. These morphological findings are complemented by hydrodynamic sizing (DLS), FTIR-derived DD%, and elemental profiling by EDX.

### DLS of synthesized ChNP

DLS analysis was employed to determine the hydrodynamic diameter and size distribution of ChNPs synthesized from *L. vannamei* shells. As shown in Table 2, the average particle diameter measured by cumulant analysis was 641.5 nm at 25°C in a medium with a viscosity of 0.8878 cP. The polydispersity index (PDI) of 0.285 indicates a moderately narrow distribution, reflecting relatively good homogeneity. This supports the effectiveness of the ionic gelation method using sodium tripolyphosphate (TPP) in producing stable nanoscale particles.

**Table 2:** Dynamic light scattering analysis of chitosan nanoparticles

Parameter	Value	Unit	Condition
Mean hydrodynamic diameter	641.5	nm	25°C
Polydispersity Index	0.285	–	–
Temperature	25	°C	Fixed
Viscosity of the Medium	0.8878	cP	Water at 25°C

Notably, the DLS-derived diameter is larger than the SEM-based size due to the hydration shell captured by DLS, as expected. These results are consistent with the SEM observations, which revealed compact, spherical particles with minimal aggregation. Across 24 h of stability testing, aggregation indices (AI = d_24_/d_0_) remained within the stability criterion (AI ≤ 1.2) for pH 5.5 (0–50 mM NaCl) and pH 7.0 (0 mM NaCl), while mild aggregation (AI > 1.2) was observed at pH ≥ 7.0 with 100 mM NaCl. Zeta potentials remained ≥ +18 mV under most conditions, confirming high colloidal stability. Together, the DLS and SEM findings confirm the structural integrity and dispersibility of ChNP, reinforcing their potential for biomedical and aquaculture applications.

### FTIR spectroscopy

**Figure 3:**
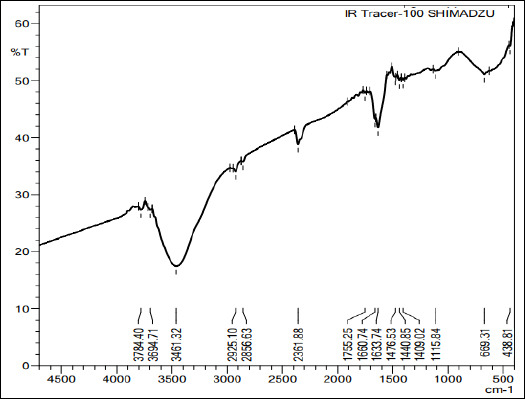
The Fourier transform infrared spectroscopy spectrum of nanochitosan synthesized from *Litopenaeus vannamei* shrimp shells indicates the presence of characteristic –OH, –NH_2_ –CH, and P=O functional groups. The prominent peak at 1115.84 cm^-¹^ confirms the interaction of TPP with crosslinking.

FTIR spectroscopy was performed to characterize the functional groups and confirm the ionic interaction between chitosan and sodium tripolyphosphate (TPP) in the synthesized ChNP. As shown in Figure 3, the broad band at 3784–3461 cm^-¹^ corresponds to O–H/N–H stretching, the peaks at 2925 and 2856 cm^-¹^ indicate aliphatic C–H stretching, the band at 1660–1634 cm^-¹^ reflects amide I/residual acetyl groups, and a distinct 1115 cm^-¹^ peak evidences P=O stretching from TPP; minor bands at 669 and 439 cm^-¹^ correspond to N–H out-of-plane bending. These features indicate that ionic gelation retained key chitosan functionalities without major backbone alteration. The chitosan precursor showed a DD of 97.8 ± 0.2% (mean ± SD, n = 3), which was calculated from the A1655/A3450 ratio using a standard calibration, supporting the use of highly deacetylated, biocompatible-grade chitosan.

### Inhibition of the formation of the initial biofilm: adherent cell biomass (OD595 nm)

**Figure 4:**
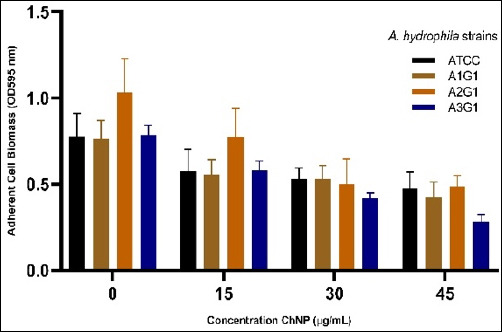
Concentration-dependent inhibition of adherent cell biomass (OD_595_) in four *Aeromonas hydrophila* strains (ATCC, A1G1, A2G1, and A3G1) treated with increasing concentrations of chitosan nanoparticles. Data are expressed as mean ± standard deviation from three independent replicates.

This study aimed to evaluate the inhibitory effect of shrimp-shell–derived ChNP on the early-stage biofilm formation of various *A. hydrophila* strains, which was quantified via the crystal violet adherence assay (OD595 nm). We hypothesized that ChNP would significantly reduce adherent cell biomass in a dose-dependent manner due to its cationic and nanoscale nature. Our findings confirmed this hypothesis. As shown in Figure 4, all tested strains exhibited a marked reduction in biofilm formation upon ChNP treatment, with strain A3G1 showing the most significant inhibition, particularly at 45% concentration, with a reduction from 0.787 ± 0.044 to 0.317 ± 0.041 OD595 units (p < 0.05). This corresponds to >59% inhibition, calculated using the inhibition formula detailed in the Materials and Methods section. Statistical analysis using Tukey’s HSD post hoc test confirmed that the observed reductions were significant across concentrations and among different strains, with the most pronounced difference identified between the highly responsive strain A3G1 and the less responsive strain A2G1, in agreement with the phenotypic variability reported by Mohamed *et al*. [28] and Zhao *et al*. [29].

### Planktonic cell growth inhibition (OD600 nm)

The ANOVA revealed that both the *A. hydrophila* strain and ChNP concentration had a statistically significant effect on planktonic cell growth inhibition. Notably, increasing ChNP concentrations (15%, 30%, and 45%) resulted in a consistent and significant reduction in OD600 values across all tested strains (p < 0.05).

**Figure 5:**
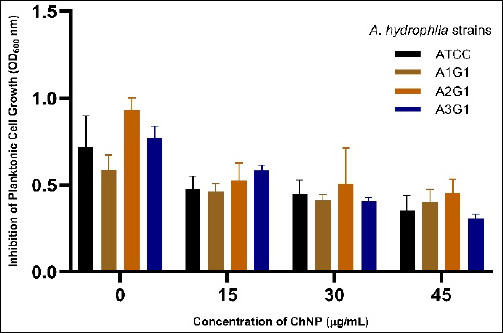
Concentration-dependent inhibition of planktonic cell growth (OD_600_) in four *Aeromonas hydrophila* strains (ATCC, A1G1, A2G1, and A3G1) treated with increasing doses of chitosan nanoparticles. Data are expressed as mean ± standard deviation of triplicate assays.

As illustrated in Figure 5, the mean OD600 decreased from 0.7188 ± 0.1794 (ATCC, 0% ChNP) to 0.3548 ± 0.0848 at 45% ChNP, indicating dose-dependent inhibition. Strain A2G1 exhibited the highest OD600 at 0% ChNP (0.931 ± 0.1480), suggesting strong planktonic growth in the absence of treatment. However, the steepest drop in OD600 values was observed at 45% (0.452 ± 0.0368), reflecting a substantial inhibitory response.

Although A3G1 also demonstrated notable reductions, the Tukey’s HSD test showed that the greatest difference occurred between the 0% and 45% concentrations across all strains (p = 0.0000). Furthermore, a two-way ANOVA revealed a significant interaction between strain and concentration (p = 0.0044), indicating strain-specific responses to ChNP exposure.

### Biofilm degradation assay of mature biofilms (OD595 nm)

**Figure 6:**
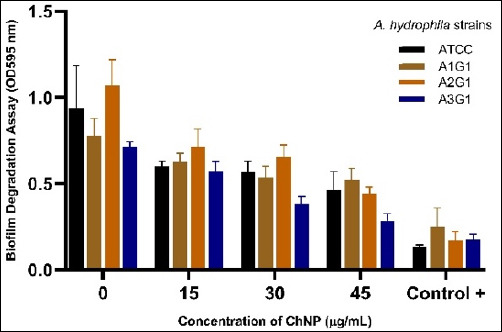
Concentration-dependent degradation of preformed biofilms in four *Aeromonas hydrophila* strains (ATCC, A1G1, A2G1, and A3G1) exposed to varying concentrations of chitosan nanoparticles. OD_595_ values indicate residual biofilm biomass. The positive control group (Control +) represents biofilm inhibition by standard antibiotic treatment. Data are presented as mean ± standard deviation from triplicate independent experiments.

This study examined the degradative effects of ChNPs against mature *A. hydrophila* biofilms by measuring residual biomass through OD595 readings. As shown in Figure 6, all four strains (ATCC, A1G1, A2G1, and A3G1) exhibited declining OD595 values with increasing ChNP concentrations (0–45 μg/mL).

Among them, A3G1 showed the greatest reduction, with OD595 decreasing from 0.717 ± 0.021 at 0% to 0.264 ± 0.033 at 45%, approaching the level observed in the positive control group (OD595 ≈ 0.174 ± 0.032). Two-way ANOVA confirmed that both strain type (p < 0.0001) and ChNP concentration (p < 0.0001) had statistically significant main effects, as well as a significant interaction effect (p < 0.001).

Pairwise Tukey’s HSD test further confirmed that A1G1 and A3G1 showed statistically significant reductions in biofilm at both 30% and 45% ChNP concentrations compared with the untreated control (p < 0.05). For example, the mean difference in OD595 between A1G1_0 and A1G1_45 was −0.2578 (p = 0.0027), while A3G1_0 versus A3G1_45 showed an even greater reduction (−0.446, p < 0.001). In contrast, intermediate concentrations (e.g., 15%) yielded non-significant reductions in several strains.

### Antibiofilm responses by strain

After a comprehensive evaluation of three key biofilm development stages, namely, adherent cell biomass (OD595), planktonic growth (OD600), and mature biofilm degradation (OD595), the A3G1 strain demonstrated the most consistent and pronounced sensitivity to ChNP treatment.

The inhibition percentage exceeded 59% during early adhesion and 63% in the degradation of mature biofilms, indicating a clear concentration-dependent pattern. A3G1 was selected for a detailed quantitative comparison due to its robust, reproducible response across assays. Table 3 summarizes the inhibitory effects across ChNP concentrations, highlighting statistically significant reductions (p < 0.05) in all tested parameters.

**Table 3:** Significant inhibitory effects of chitosan nanoparticles (ChNP) (p < 0.05) on *Aeromonas hydrophila* A3G1 biofilm dynamics

ChNP concentration (%)	Adherent biomass (OD595 nm)	Adherent inhibition (%)	Planktonic growth (OD600 nm)	Planktonic inhibition (%)	Biofilm degradation (OD595 nm)	Biofilm degradation (%)
0	0.787 ± 0.044	–	0.770 ± 0.033	–	0.717 ± 0.021	–
15	0.618 ± 0.052	21.5%	0.568 ± 0.031	26.2%	0.529 ± 0.037	26.2%
30	0.452 ± 0.048	42.6%	0.390 ± 0.029	49.4%	0.361 ± 0.035	49.7%
45	0.317 ± 0.041	59.7%	0.283 ± 0.030	63.2%	0.264 ± 0.033	63.2%

These results underscore the strain-specific antibiofilm efficacy of ChNPs and their potential for targeted biocontrol in aquaculture.

## DISCUSSION

### Phenotypic and molecular characterization of *A. Hydrophila* isolates

The isolates analyzed in this study were biochemically consistent with *A. hydrophila*, exhibiting defining enzymatic profiles (catalase, oxidase, indole, urease, and VP-positive) and characteristic sugar fermentation patterns. Minor deviations in the activity of citrate and lysine decarboxylase reflected normal intra-species variability [1, 4, 30]. Resistance to ampicillin and sensitivity to O/129 further substantiated species-level identification [31, 32].

SEM imaging confirmed typical rod-shaped morphology, while 16S rRNA sequencing (>99% similarity) validated molecular identity and pathogenic relevance in aquaculture contexts [33, 34].

### Physicochemical and structural features of ChNP

ChNP synthesized from *L. vannamei* exhibited a spherical morphology with minimal aggregation (SEM 416 nm; DLS 641 nm). FTIR spectroscopy confirmed the preservation of N–H, O–H, amide I, and P=O bands, indicating stable ionic crosslinking with TPP and structural integrity under synthesis conditions.

Collectively, these properties demonstrate the morphological stability and functional readiness of ChNPs for biological interaction, establishing a physicochemical foundation for their subsequent antibiofilm activity [35–41].

### Antibiofilm efficacy of shrimp-shell–derived ChNPs

Shrimp-shell–derived ChNPs display broad-spectrum antibiofilm activity against *A. hydrophila*, effectively targeting adherent biomass, planktonic growth, and mature biofilms [42–48]. Strain A3G1 showed the greatest response to ChNP exposure.

The inhibition of early biofilm formation, characterized by a substantial reduction in adherent biomass at the highest concentration, likely stems from electrostatic interactions between chitosan’s protonated amino groups and anionic cell wall or extracellular polymeric substances (EPS) biofilm components, resulting in membrane destabilization and weakened surface adhesion [49–52].

The nanoscale size and enhanced surface reactivity of ChNPs further modify substratum properties, reducing hydrophobicity and bacterial affinity [53–55], while enabling early interference with quorum-sensing pathways [56–61]. These complementary mechanisms produce a concentration-dependent suppression of biofilm initiation consistent with prior findings on chitosan-mediated membrane perturbation, DNA interference, and EPS disruption [62–67].

### Mechanistic interpretation of growth and biofilm inhibition

The observed growth inhibition in A3G1, reflected by a progressive decline in OD600, and partial tolerance in A2G1 at lower concentrations, align with differences in membrane permeability and metabolic robustness [68, 69]. ChNPs are known to trigger reactive oxygen species (ROS) accumulation and adenosine triphosphate depletion, disrupting bioenergetics and quorum-sensing systems [43, 56–58, 63, 69, 70].

Supporting this interpretation, Xiao and Koo [64] described early EPS-enriched microcolony development, in which EPS constitutes up to 90% of the organic biofilm carbon [63, 71]. Subhaswaraj *et al*. [68] similarly reported 84% inhibition at 500 µg/mL ChNP, which was attributed to nanoparticle penetration into the Gram-negative envelope and downregulation of EPS or quorum-sensing genes [72, 73–76].

Together, these findings substantiate a multifactorial inhibition process involving membrane permea-bilization, ROS-mediated metabolic collapse, and EPS destabilization, thereby obstructing the early establishment of biofilms. A3G1’s greater susceptibility compared with A2G1’s partial tolerance reflects inherent differences in biofilm-regulating and quorum-sensing circuits (luxS, aerA, ahp, and ompA) [41, 60–64], interpreted here contextually without introducing new molecular claims.

### Charge-mediated disruption model

Consistent with a charge-mediated mechanism, the concentration-dependent inhibition of adherent biomass, suppression of planktonic growth, and degradation of mature biofilms, most evident in strain A3G1, suggest that protonated amino groups on ChNPs electrostatically interact with negatively charged bacterial envelopes and EPS, promoting membrane perturbation and matrix destabilization.

The positive ζ-potential of the nanoparticles supports these electrostatic interactions, favoring attraction and penetration within EPS-rich matrices. A3G1 displayed progressive OD600 reduction, whereas A2G1 showed marked suppression only at elevated concentrations despite higher basal growth.

In mature biofilms, OD595 values declined markedly at the highest ChNP concentration, approaching the positive control; two-way ANOVA indicated significant effects of both strain and concentration, as well as their interaction. Although direct visualization assays (e.g., ROS quantification, live/dead imaging, EPS staining) were not performed, the consistent concentration–response across all phases aligns with recognized electrostatic disruption models for chitosan nanomaterials and provides a coherent mechanistic explanation supported by the present dataset [56–60, 77].

### Experimental constraints and methodological limitations

All experiments were conducted under static *in vitro* conditions, with neither tank/recirculating aquaculture systems (RAS) pilots nor in vivo validation. Dosing was initially expressed as percentage dilutions (15%–45%), constraining cross-study comparability despite subsequent harmonization to mass-based units.

The vehicle and positive control parameters were not fully standardized across assays, and the replication emphasized technical repeats without a priori power analysis. Physicochemical profiling confirmed primary features (DLS, ζ-potential, SEM, and FTIR) but did not report explicit ζ-potential values (mV), chitosan DD%), batch-to-batch reproducibility, or pH/ionic-strength effects; OD595 crystal violet readings were not calibrated to biomass.

Mechanistic support was indirect, and ROS assays, membrane integrity/leakage tests, live/dead or colony-forming units, EPS carbohydrate/protein measurements, microscopy of treated biofilms (SEM/confocal laser scanning microscopy [CLSM]), ζ-shift upon exposure, and qPCR of quorum-sensing/biofilm and resistance genes were not performed. Statistical reporting emphasized p-values without effect sizes, CI, or assumption checks; IC50/regression modeling was not applied.

Ecotoxicity/cytocompatibility and environmental fate were not assessed, nor were practical deployment parameters for aquaculture (dose/frequency; feed/probiotic compatibility) defined, nor were strain-specific differences (A3G1 vs. A2G1).

### Implications and future perspectives

Within the scope of *in vitro* experimentation, shrimp-shell–derived ChNPs demonstrated potent antibiofilm action against wild-type *A. hydrophila*, underscoring their potential for biofilm management in tropical aquaculture systems [78].

The biodegradable, biopolymeric nature of chitosan, derived from shrimp-shell waste, situates this approach within a circular bioeconomy and aligns with One Health principles aimed at reducing antibiotic dependency [79].

Future pilot-scale validations, such as biofilter or recirculating system trials, and comparative benchmarking with other nanoparticle formulations will further clarify performance and ecological safety, while targeted molecular assays may elucidate strain-specific responses’ mechanistic basis.

## CONCLUSION

This study demonstrated that shrimp-shell–derived ChNP synthesized through ionic gelation exhibits a stable physicochemical profile, spherical morphology (SEM: 416 nm; DLS: 641 nm), moderate dispersity (PDI ≈ 0.28), and preserved functional groups (N–H, O–H, amide I, and P=O), supporting its readiness for biological application. The isolates recovered from diseased gourami were biochemically and molecularly confirmed as *A. hydrophila*, with >99% 16S rRNA sequence similarity to international reference strains. The synthesized ChNPs showed significant antibiofilm activity across multiple biofilm developmental stages, including the inhibition of initial adhesion, suppression of planktonic growth, and degradation of mature biofilms. Among the tested strains, A3G1 exhibited the highest responsiveness, showing >59% reduction in adherent biomass and >63% degradation of established biofilms at 45 µg/mL. These effects were statistically significant (p < 0.05) and concentration-dependent, confirming the reproducibility of ChNP activity across replicates.

The results highlight the potential of ChNPs as a biodegradable, non-antibiotic alternative for biofilm control in aquaculture. By targeting cell adhesion, EPS integrity, and quorum-sensing pathways, ChNPs could mitigate *A. hydrophila* colonization on tank surfaces, biofilters, and aquatic equipment, critical points for infection transmission. Integration into RAS or feed supplements could support disease prevention while minimizing antimicrobial resistance risks, aligning with One Health and sustainable aquaculture principles.

This work combines biochemical, molecular, and nanoscale analyses to establish a complete link between bacterial pathogenicity and nanoparticle-mediated inhibition. The simultaneous assessment of planktonic and sessile phases under controlled conditions provides a comprehensive understanding of ChNP activity. The study also validates eco-friendly chitosan utilization derived from crustacean waste, demonstrating a circular bioeconomy approach to antimicrobial innovation.

The findings are based solely on static *in vitro* assays without flow-based or in vivo validation. ROS generation, membrane leakage, EPS quantification, or gene-expression profiling were not conducted; therefore, mechanistic interpretations remain inferential. ζ-potential, DD%, and cytocompatibility data were not fully quantified, limiting cross-comparability. Statistical analyses did not include regression or dose–response modeling, and environmental toxicity or biocompatibility under aquaculture conditions was not evaluated.

Future investigations should include mechanistic assays (ROS production, EPS disruption, qPCR of *luxS*, *aerA*, *ompA*, and *ahp*), live/dead imaging, and structural visualization of treated biofilms using CLSM or SEM. Pilot-scale evaluations in biofilters or RAS systems, along with *in vivo* safety and efficacy studies, are warranted to establish optimal dosing strategies, frequency, and long-term ecological safety. Comparative trials with metallic or hybrid nanoparticles could further contextualize ChNP performance within broader nanotherapeutic frameworks.

Within the scope of the present *in vitro* study, shrimp-shell–derived ChNPs effectively inhibited *A. hydrophila* biofilm formation and promoted biofilm degradation through concentration-dependent, charge-mediated interactions. These results reinforce the potential of ChNPs as a sustainable, environmentally benign biocontrol agent for the management of aquaculture biofilms. With continued refinement and *in vivo* validation, nanochitosan could emerge as a practical nanobiopolymer solution contributing to antibiotic stewardship, circular bioeconomy, and aquatic health resilience under the One Health paradigm.

## AUTHORS’ CONTRIBUTIONS

RZ: Conceptualization, methodology, investigation, data curation, formal analysis, visualization, and drafted and revised the manuscript. WT: Conceptualization, supervision, validation, resources, and drafted and edited the manuscript. JR: Investigation, data curation, and drafted and edited the manuscript. EBA: Conceptualization, supervision, validation, and edited the manuscript. MY: Investigation, resources, and drafted and edited the manuscript. MAA: Methodology, project administration, validation, and drafted and edited the manuscript. SK: Methodology, validation, visualization, and drafted and edited the manuscript. PDWS: Data curation, visualization, and drafted and edited the manuscript. AS: Validation, resources, and drafted and edited the manuscript. SS: Conceptualization, supervision, project administration, resources, and drafted and edited the manuscript. All authors have read and approved the final version of the manuscript.
